# Precision of point of care glucose metre measurements in the context of neonatal hypoglycemia

**DOI:** 10.1093/pch/pxae049

**Published:** 2024-08-20

**Authors:** Julie L V Shaw, Saranya Arnoldo, Sukhbir Kaur, Michael J Knauer, Felix Leung, Heather Paul, Vinita Thakur, Davor Brinc

**Affiliations:** Division of Biochemistry, The Ottawa Hospital and Eastern Ontario Regional Laboratories Association, Ottawa, Ontario, Canada; Department of Pathology and Laboratory Medicine, University of Ottawa, Ottawa, Ontario, Canada; Department of Laboratory Medicine and Pathobiology, Laboratory Medicine and Genetics Program, Trillium Health Partners, University of Toronto, Toronto, Ontario, Canada; Division of Clinical Biochemistry, Saskatchewan Health Authority, Saskatoon, Saskatoon, Saskatchewan, Canada; Department of Pathology and Laboratory Medicine, London Health Sciences Centre, University of Western Ontario, London, Ontario, Canada; Department of Pathology and Laboratory Medicine, Sinai Health System, University of Toronto, Toronto, Ontario, Canada; Department of Laboratory Medicine and Pathobiology, University of Toronto, Toronto, Ontario, Canada; Alberta Precision Laboratories, Calgary, Alberta, Canada; Department of Pathology and Laboratory Medicine, University of Calgary, Canada; Newfoundland and Labrador Health Services, Pathology and Laboratory Medicine Program, Memorial University of Newfoundland, St. John's, Newfoundland and Labrador, Canada; Department of Laboratory Medicine and Pathobiology, University of Toronto, Toronto, Ontario, Canada; Laboratory Medicine Program, University Health Network, University of Toronto, Toronto, Ontario, Canada

**Keywords:** Hypoglycemia, Imprecision, Neonatal, Point of care testing, POCT

Point of care testing (POCT) for capillary blood glucose is commonly used in the neonatal population (<28 days old) to guide hypoglycemia treatment. The Canadian Pediatric Society recommends treatment of hypoglycemia in neonatal patients when the blood glucose concentration is (i) <2.6 mmol/L in the first 72 h or (ii) <3.3 mmol/L after 72 h of life (1). The accuracy of laboratory measurements is dependent on the method’s bias and precision. Bias refers to the closeness of a measured value to the true value. Our previous study demonstrated the expected bias for POCT glucose measurements compared to central laboratory measurements for two hospital-approved devices. The Roche Inform II metre showed a positive bias of approximately 0.3 mmol/L and the Nova StatStrip showed a negative bias of approximately 0.4 mmol/L compared to the central laboratory for glucose measurements <3.0 mmol/L (2). Here, we present follow-up data, illustrating the imprecision of POCT glucose metres based on real clinical repeat measurements. Precision is the ability to replicate the same result with repeat measurements of the same sample and is typically expressed as the coefficient of variation (CV), which is the standard deviation (SD) divided by the mean value, expressed as a percent. Our data demonstrate the expected variability with repeat POCT glucose measurements, which can help clinicians determine if observed differences are clinically significant or due to inherent metre imprecision.

## METHODS

Repeat POCT glucose results from neonates were obtained from five hospital sites in Ontario, including The Ottawa Hospital (n = 98), London Health Sciences Centre (n = 100), Trillium Partners (n = 340), William Osler Health (n = 119), and Lakeridge Health (n = 89). All repeat measurements were performed on the same patient within 2 min. Seven hundred and forty-six repeat measurements were identified, from two models of POCT glucose metre, the Nova Stat Strip (n = 548) and the Roche Accuchek Inform II (n = 198).

Data were categorized into three bins based on the initial POCT measured glucose concentration (<2.6 mmol/L, 2.6 to 3.3 mmol/L, >3.3 mmol/L). The SD was calculated for each set of repeats along with the CV (SD divided by the mean, expressed as a percentage). The average CV for all repeat measurements was calculated for each concentration bin.

## RESULTS

**Figure 1.**
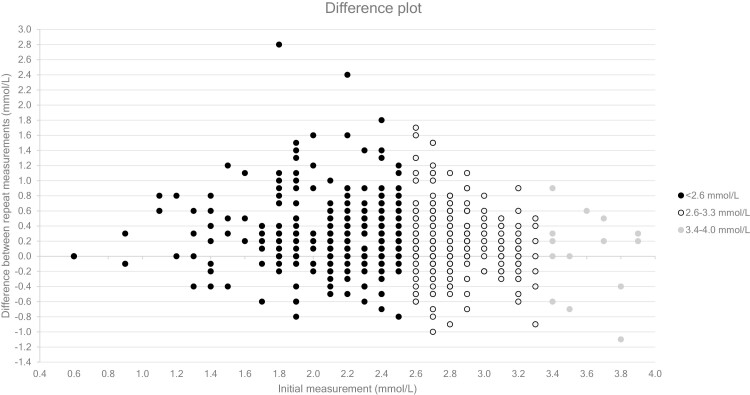
Difference plot showing the initial measurement on the x-axis and difference between initial and repeat measurement on type y-axis. Initial measurements <2.6 mmol/L are shown in black, initial measurements 2.6 to 3.3 mmol/L are shown in white with black outline, and initial measurements 3.4 to 4.0 mmol/L are shown in grey. Data for initial measurements >4.0 mmol/L are not included to allow for better visualization of the data for a lower initial concentration range

Differences between repeat measurements <4.0 mmol/L are shown in Figure 1. The mean CV for repeat glucose measurements between <2.6 mmol/L (n = 390) was 7.4% (95% CI 6.7 to 8.1), for measurements 2.6 to 3.3 mmol/L (n = 297) was 5.5% (CI 4.9 to 6.0) and for measurements >3.3 mmo/L (n = 59) was 3.9% (CI 2.9 to 4.9). Repeat measurements were most often equivalent or higher when the initial measurement was ≤3.3 mmol/L (542/687). No consistent trend was for initial measurements >3.3 mmol/L.

## DISCUSSION

Here, we highlight the imprecision of POCT glucose measurements based on repeat measurements in neonatal patients across two models of metres from five hospital sites. Our previous work demonstrated imprecision of approximately 6% for glucose concentrations around 2.5 mmol/L, based on quality control measurements. Here, the imprecision of repeat measurements with patient samples is similar to what was seen with repeat measurements of quality control material for glucose concentrations 2.6 to 3.3 mmol/L. Our data suggests that when an initial POCT glucose measurement of 2.6 mmol/L is repeated within 2 min of the first measurement, the repeat measurement could be anywhere from 2.5 to 2.7 mmol/L, based on one SD. A repeat of an initial measurement of 3.3 mmol/L within 2 min could yield a result between 3.1 and 3.5 mmol/L, based on one SD. Thus, when repeat POCT measurements are made, a repeat glucose measurement different from the initial glucose measurement may not represent a true difference if the result is within two SDs (11%) based on expected imprecision. The Canadian Standards Association recommends imprecision of no more than 12.5% and 7.5% for glucose <2.5 mmol/L and ≥2.5 mmol/L, respectively, for diabetes management (3). It is important for clinicians to interpret repeat measurement values in the context of expected differences based on inherent imprecision.

Some limitations of our study relate to not knowing what occurred between the initial and repeat measurements. Was the repeat measurement made using a fresh capillary specimen? Did treatment occur between repeats? Lastly, the reason for performing a repeat measurement is unknown. It is possible the repeat measurement was initiated due to an error associated with the first measurement.

In summary, we recommend that physicians caring for neonatal patients consider the following:

1. Understanding glucose metre precision and bias and discussing this with the local laboratory.
2. Keeping in mind the inherent imprecision of POCT metres when interpreting repeat measurements to help determine whether a change is real.
3. Sending specimens to the laboratory for confirmatory measurements or when POCT results do not fit clinically, rather than ordering repeat POCT measurements.
